# Advances in Additive Manufactured Scaffolds Mimicking the Osteochondral Interface

**DOI:** 10.1002/anbr.202400059

**Published:** 2024-09-30

**Authors:** Ivo A. O. Beeren, Pieter J. Dijkstra, Carlos Mota, Sandra Camarero-Espinosa, Matthew B. Baker, Lorenzo Moroni

**Affiliations:** Department of Complex Tissue Regeneration, MERLN Institute for Technology-Inspired Regenerative Medicine, Maastricht University, Universiteitssingel 40, Maastricht 6229ER, The Netherlands; Department of Complex Tissue Regeneration, MERLN Institute for Technology-Inspired Regenerative Medicine, Maastricht University, Universiteitssingel 40, Maastricht 6229ER, The Netherlands; POLYMAT, University of the Basque Country UPV/EHU, Tolosa Avenue 72, Donostia/San Sebastián 20018, Spain; IKERBASQUE Basque Foundation for Science, Plaza Euskadi 5, Bilbao 48009, Spain; Department of Complex Tissue Regeneration, MERLN Institute for Technology-Inspired Regenerative Medicine, Maastricht University, Universiteitssingel 40, Maastricht 6229ER, The Netherlands

**Keywords:** additive manufacturing, dynamic materials, gradients, osteochondral regeneration

## Abstract

Architectural, compositional, and mechanical gradients are present in many interfacial tissues in the body. Yet desired for regeneration, the recreation of these complex natural gradients in porous scaffolds remains a challenging task. Additive manufacturing (AM) has been highlighted as a technology to fabricate constructs to regenerate interfacial tissues. Integration of different types of gradients, which can be physical, mechanical, and/or biochemical, shows promise to control cell fate and the regeneration process in a spatial controlled manner. One of the most studied tissue interfaces is the osteochondral unit which connects cartilage to bone. This tissue is often damaged because of trauma or ageing, leading to osteoarthritis; a degenerative disease and a major cause of disability worldwide. Therefore, in view of osteochondral (OC) regeneration, a state-of-the-art overview of current approaches is presented to manufacture gradient scaffolds prepared by AM techniques. The focus is on thermoplastic, hydrogel, and hybrid scaffolds comprising gradients that induce biomimicry by their physical and biological properties. The effect of these different systems on OC tissue formation in-vitro and in-vivo is addressed. Finally, an outlook on current trends of dynamic materials is provided, including proposals on how these materials could improve the mimicry of scaffolds applied for OC regeneration.

## 1 Introduction

In tissue engineering (TE), researchers aim to create biomimetic constructs that support restoring or replacement of (damaged) tissues.^[1]^ To this end, biomaterials— 3natural or synthetic materials that interact with a biological system^[2]^—have been processed into scaffolds that can be implanted and direct the behavior of migrated or pre-seeded cells in a controlled fashion. The biomaterial to be used depends on the intended application, and its properties are aimed at mimicking the features of the native tissue. As diseases or damages rarely affect a single type of tissue, one of the main challenges in TE is the design and preparation of constructs covering the changes in properties at tissue interfaces. At tissue interfaces, gradients—defined as a stepwise or gradual transition of a certain tissue characteristic, molecular concentration, or physical or biological property—are typically present. Four types of gradients are distinguished: cellular, compositional, architectural, and mechanical gradients.^[3]^

**Figure 1:**
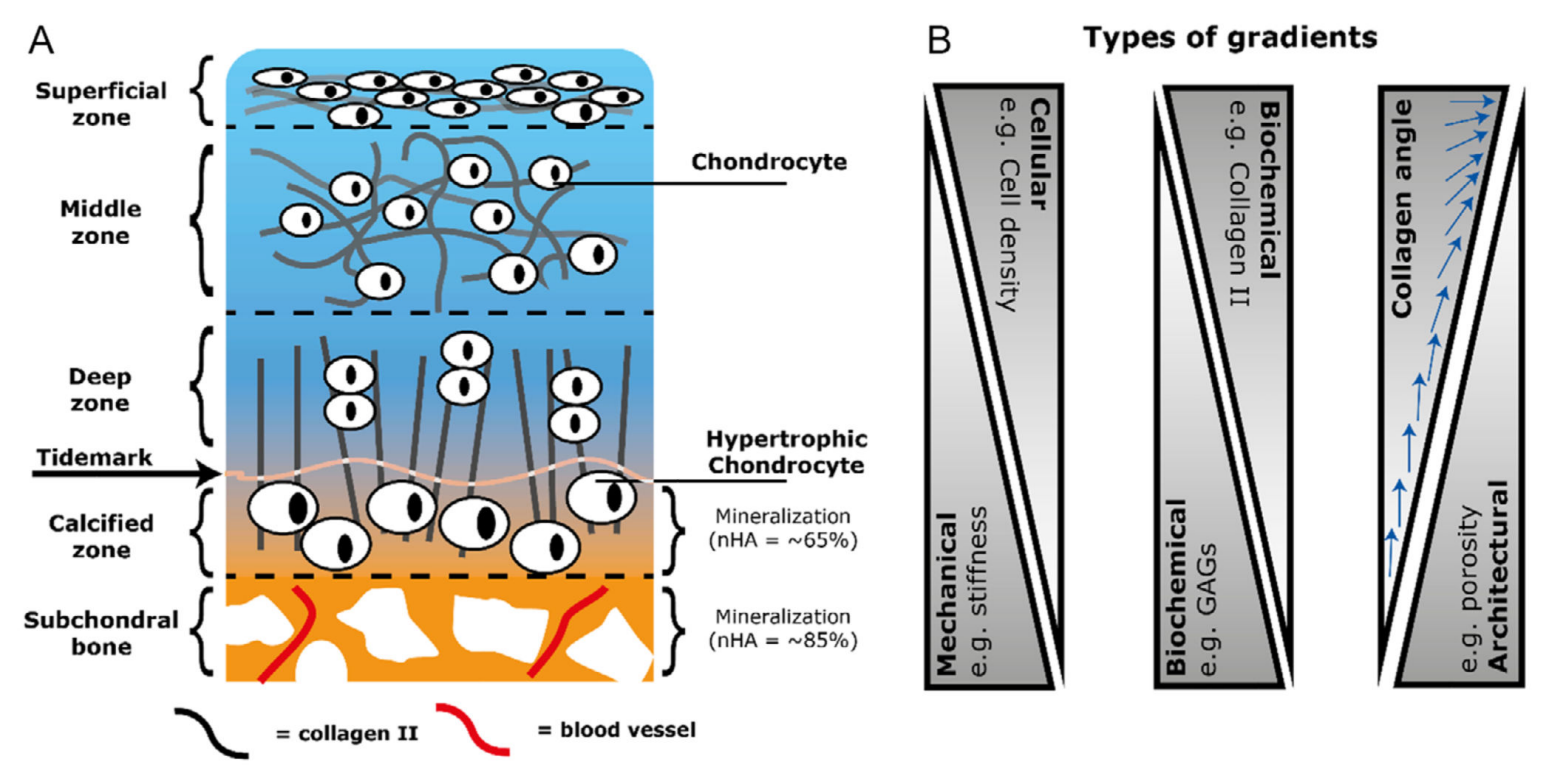
A schematic of the OC unit. A) The OC unit is represented as a stratified tissue comprising a superficial, middle, deep, calcified cartilage, and subchondral bone zone. Each zone contains a (slightly) different cell type and has a different biochemical composition. B) Across the unit, different types of gradients are found. Raman spectroscopy was used to visualize the presence of (countercurrent) cellular (e.g., cell density), biochemical (e.g., GAGs or hydroxyapatite), architectural (e.g., the collagen fiber orientation), and mechanical gradients (e.g., the elastic modulus).^[8]^

The musculoskeletal system comprises cartilage to bone, tendon to bone, and ligament to bone tissue interfaces. The osteochondral (OC) unit, the cartilage to bone interface, is one of the most studied tissue interfaces, and contains all four types of gradients mentioned previously.^[4–7]^ The unit is often schematically depicted as a zonal-like tissue, comprising several gradients from the superficial zone toward the subchondral bone (Figure 1A,B). For example, oval-shaped chondrocytes located in the superficial zone gradually become hypertrophic, and finally become osteogenic cells in the subchondral bone region. The cell density decreases concomitantly with the change in phenotype. Associated with the cellular gradient is the compositional change in extracellular matrix (ECM); from the superficial layer toward the calcified layer, the presence of collagen II decreases and the glycosaminoglycan (GAG) and collagen X content increases (Figure 1B).^[8]^ From the onset of mineralization—the tidemark line – the dry weight of the inorganic hydroxyapatite content gradually increases from 65.1 ± 2.3% in the calcified zone to 85.8 ± 3.4% in the subchondral bone.^[9]^ In addition to biochemical and cellular gradients, architectural gradients are observed in the OC tissue. For example, the collagen orientation changes from parallel to the joint plateau at the cartilage surface toward perpendicular in the deep zone.^[8]^ The porosity significantly changes between the cortical bone (5%–15%) and subchondral (trabecular) bone (>40%).^[10,11]^ As a result of the biochemical and architectural gradients, a mechanical gradient arises between the superficial cartilage (0.24 ± 0.05 MPa)^[12]^ and sub-chondral bone (3.9 ± 1.5 GPa).^[13]^

The implementation of gradients in OC scaffolds has garnered a lot of attention in the last decades.^[7,14,15]^ Currently, additive manufacturing (AM) is highlighted as the most promising technique to fabricate scaffolds for OC regeneration.^[16–18]^ In this review, the most applied AM technologies for scaffold preparation in OC tissue regeneration are presented. Consecutively, we summarize the use of AM to fabricate scaffolds containing different types of gradients, in view of OC regeneration. Many thermoplastic and hydrogel materials have been studied for OC applications.^[16,19]^ Yet, these materials typically do not adapt to environmental cues, while adaptability is an important characteristic of the native ECM. Hence, advances in the development of dynamic biomaterials—materials that contain reversible bonds—that have been successfully applied in AM applications are reviewed. We discuss how the integration of these materials in AM constructs may contribute to mimicking features of the OC tissue.

## 2 AM Technologies

Biofabrication is currently defined as “the automated generation of biologically functional products with structural organization from living cells, bioactive molecules, biomaterials, cell aggregates such as micro-tissues, or hybrid cell-material constructs, through bioprinting or bioassembly and subsequent tissue maturation processes”.^[20]^ A comprehensive description of AM technologies and associated terminology in biofabrication strategies was recently reviewed.^[21]^ In this section, a more detailed description of the most common AM technologies used for the fabrication of OC scaffolds comprising gradients is provided. These technologies can be classified into three categories: material extrusion (e.g., melt-extrusion-based AM, melt electro-writing, 3D (bio)plotting, microfluidic printing), vat photopolymerization (e.g., digital light processing, stereolithography), and powder bed fusion (e.g., selective laser melting and selective laser sintering). When a biomaterial is selected, the intrinsic (e.g., rheological and thermal) properties must be compatible with a specific AM technique to enable the deposition fibers into stable scaffolds.^[22]^ Simultaneously, the biological properties should direct the behavior of cells toward deposition of the intended tissue. Another consideration in the selection of a biomaterial is either to use a biopolymer (e.g., a protein or polysaccharide) or a synthetic material. The advantage of using biopolymers is their intrinsic biocompatibility, but a common issue is batch-to-batch variations in isolated products that hinder reproducibility in AM as well as cellular output. Contrary, fully synthetic materials are often well-defined due to control over synthesis and final composition, but often lack intrinsic biological activity. Both types of materials may require additional chemical modification to improve their applicability. For example, hyaluronic acid (HA), a natural polymer found in joints, requires stabilization by, for example, crosslinking to avoid rapid degradation. Synthetic polymers generally need to be modified to introduce addressable groups that allow grafting of biomolecules. Selecting a biomaterial that possesses the suitable biological as well as printing properties remains currently a challenge.

### 2.1 Extrusion-Based AM

**Figure 2:**
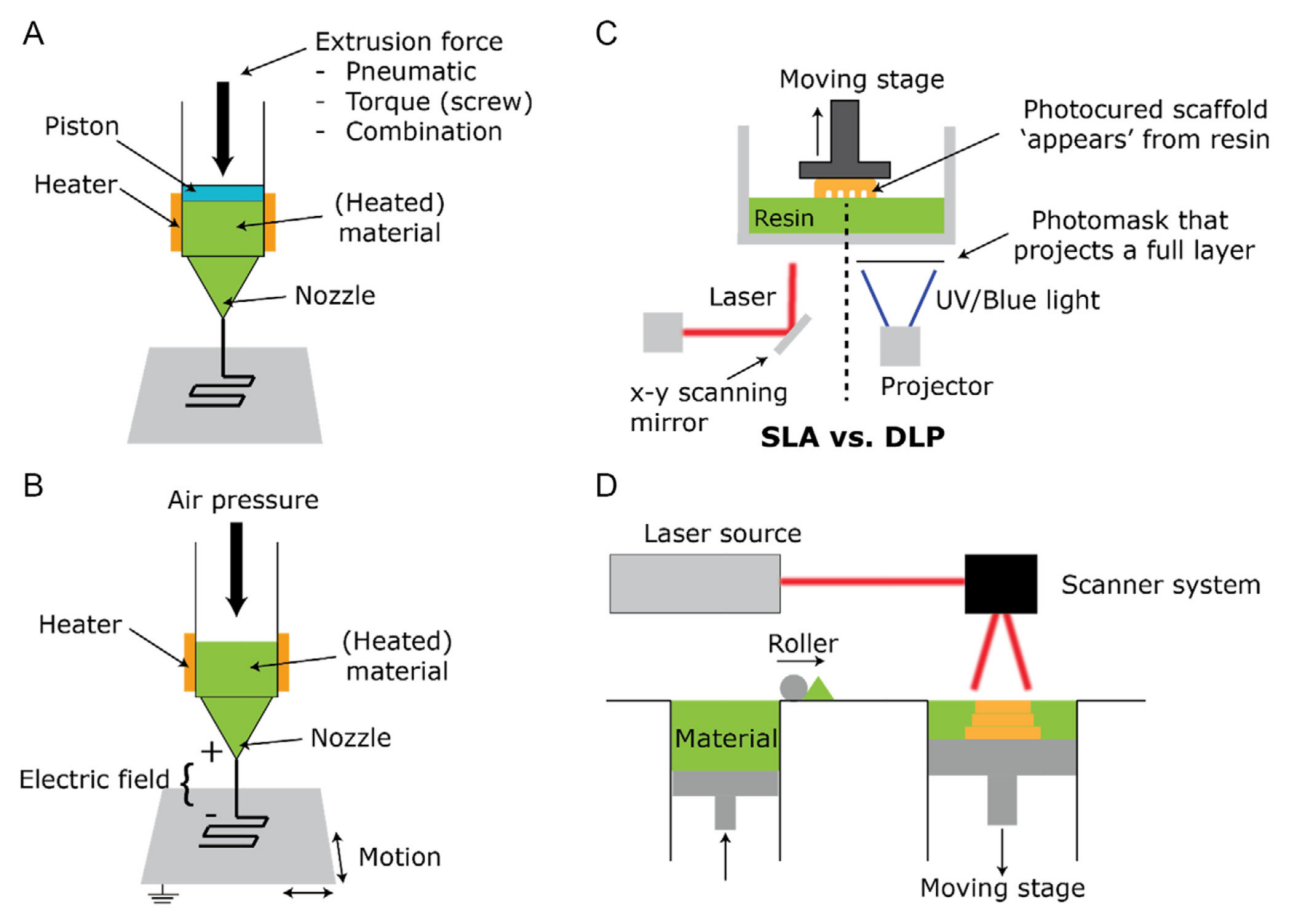
An overview of the most applied AM technologies for the fabrication of OC scaffolds. A) Extrusion-based deposition, B) Melt electrowriting, C) Vat photo-polymerization (stereolithography versus digital light processing), and D) Powder bed fusion are depicted.

In extrusion-based AM, either hydrogels or thermoplastic polymeric (composite) materials are extruded through a nozzle using torque, pressure, or a combination of both into a porous 3D construct (Figure 2A). Extrusion-based AM is the most used technology to fabricate OC scaffolds for tissue engineering applications.^[16]^ This technique uses computer aided design (CAD) models that enable the controlled deposition of fibers.

The introduction of architectural and/or compositional gradients is possible by changing the CAD model or material reservoir, respectively.

#### 2.1.1 Melt-Extrusion-Based Techniques

In melt-extrusion-based AM techniques (ME-AM), highly viscous thermoplastic materials are heated above their melting temperature. This viscous melt is extruded through a nozzle by applying pressure, torque, or a combination of both. Upon cooling in ambient air, the deposited fiber solidifies. Here, fused filament fabrication (FFF) and 3D fiber deposition (3DF) are the most used extrusion techniques. Between the techniques, the main difference is the loading method of the polymer into the print head. In FFF, a filament of the polymer from mechanically driven rotating spool is fed, whereas in 3DF a cartridge with molten polymer pellets or particles is used. A few examples of commonly applied polymers in ME-AM for OC applications are poly(ε-caprolactone) (PCL),^[23]^ polylactic acid (PLA),^[24]^ poly(ester-urethane)s,^[25,26]^ and poly(ethylene oxide terephthalate)/poly(butylene terephthalate)s (PEOT/PBT).^[27,28]^ ME-AM scaffolds are often used to prepare scaffolds for bone regeneration applications, as the mechanical properties of the applied materials typically display a substrate stiffness beyond that of the cartilage ECM.^[29]^ High substrate stiffnesses (>10 MPa) tend to upregulate osteogenic markers such as Runt-related transcription factor 2 (RUNX2) in cell types such as mesenchymal stromal cells.^[30]^ To resemble the mineralized component of the bone ECM, these polymers can be pre-blended with inorganic materials such as tricalcium phosphate (TCP) or hydroxyapatite (HA).^[31,32]^

#### 2.1.2 3D (Bio)Plotting

In 3D plotting typically a shear-thinning polymer or hydrogel is extruded through a nozzle by applying air pressure at temperatures between 4 and 37 °C, with or without encapsulated cells. Compared to materials applied in ME-AM, hydrogels, and the materials they are prepared from, have orders of magnitude lower stiffness and are amorphous. To enable the deposition of stable 3D hydrogel-based fibers, the precursor has to display intrinsic elastic recovery behavior, or an external cue should induce swift gelation upon extrusion (e.g., temperature or UV light). The latter approach may require the chemical modification of the natural or polymeric material with crosslinkable groups such as methacrylates.^[33]^ Most employed materials are composed of natural biopolymers such as alginate,^[34,35]^ HA,^[36,37]^ chondroitin sulfate,^[38]^ collagen,^[39,40]^ and gelatin.^[41]^ In addition, synthetic poly(ethylene glycol) (PEG)-based materials have been frequently applied.^[42,43]^

When cells are encapsulated in the hydrogel precursor, the solution is called a bioink. A bioink is defined as “a formulation of cells suitable for processing by an automated biofabrication technology that may also contain biologically active components and biomaterials”.^[44]^ To this end, the hydrogel precursor can be modified to incorporate features of the native ECM in the material, directing the fate of seeded cells. However, the material properties that favor cell survival may conflict with the required properties for printing.^[45]^ For example, a denser polymer network enhances the viscosity, which increases print fidelity, but enhances shear stress exerted on encapsulated cells.^[46,47]^ The combination of bioinks via multi-cartridge deposition enables the precise patterning of cells within a scaffold, making it a powerful strategy.

#### 2.1.3 Microfluidic Printing

Microfluidic printing uses pressure to extrude liquid-like hydrogel precursors and a crosslinker through a microfluidic print head. This process permits the inclusion of cells in the precursors, forming a bioink, and enables the controlled patterning of cells. When the solutions meet, a stable fiber is formed upon crosslinking during the residence time in the nozzle. Crosslinking can be a result of, for example, temperature or pH dependent physical interactions or can be induced chemically by the application of, for example, UV light. In this technique, the design of the chip can be adapted to control the microfluidic mixing of multiple solutions. In addition, the design could also enable the extrusion of fibers containing a core–shell morphology.^[48]^

#### 2.1.4 Melt Electro-Writing (MEW)

MEW enables the deposition of multiple layers of solidified fibers with diameters commonly smaller than 100 μm; a resolution that is challenging to achieve with ME-AM and 3D plotting. Instead of relying on pressure and torque, MEW applies an electric field to draw a molten polymer from a nozzle onto a grounded collector surface (Figure 2B).^[49]^ Compared to conventional solution electrospinning, the advantage of this approach is that no (toxic) solvents have to be used. Most studies using MEW are limited to using PCL, and further technological advances are needed to broaden the diversity of its material pool.^[50]^ Since MEW produces relative thin fibers, the technique is often applied to resemble parts of the (calcified) cartilage phase, having a thickness of 2–4 mm.

### 2.2 Vat Polymerization

In vat polymerization techniques, such as stereolithography (SLA) and digital light processing (DLP), liquid photosensitive monomers and oligomers, called resins, are photochemically polymerized into specific patterns (Figure 2C). By moving the scaffold platform in an *x, y*, and *z* direction, a 3D construct emerges from the liquid resin. The viscosity of the resin should be low enough to ensure quick removal of unreacted resin in each layer during stage movement. To improve the z resolution in this technique, light attenuating additives were added to the reservoir.^[51,52]^ The additive limits undesired out-of-focal polymerization via absorption of scattered light. These techniques enable the generation of (architectural gradient) scaffolds with relative high resolution. The formation of compositional gradients is more challenging as switching of the material reservoir is not trivial. For example, DLP set-ups had to be adjusted to facilitate manual or automated switching of a material reservoir.^[53]^

The differences between SLA and DLP are related to the method to photo-crosslink each layer. In SLA, a scanning mirror focuses a laser on the top layer of a resin. In DLP, one full layer is directly projected using a photomask, which reduces the fabrication time.^[54]^ Over the last decade, cytotoxic epoxy resins have been replaced by biocompatible resins such as poly(ethylene glycol)-diacrylate (PEGDA) or gelatin-methacrylate (GelMA).^[55,56]^ Although the amount of compatible biomaterials was limited, the portfolio of biodegradable, synthetic resins, such as poly(ester) urethanes acrylates, is currently rapidly expanding.^[57,58]^ In addition to material advances, non-toxic light blocking additives were discovered that maintained the fabrication of constructs with high resolution. Grigoryan et al. demonstrated that native-like vascular structures could be fabricated from a PEGDA resin containing a biocompatible food dye as photoabsorber.^[59]^ Moreover, recent studies demonstrated that optimization of the concentration of photo-absorber, photoinitiator, and UV illumination time allows the encapsulation of viable cells in the resin.^[60]^

### 2.3 Powder Bed Fusion

Selective laser sintering (SLS) uses a directed laser to pattern a scaffold in a layer-by-layer fashion. However, instead of a liquid resin, a powder bed is used in which particles are locally fused by applying heat via a laser (Figure 2D). The powder bed stage with scaffold moves downward after each layer, and a new thin layer of powder is delivered via a roller system. The layer is sintered, and the process is repeated until a 3D construct is eventually formed.^[61]^ In this technique, thermoplastic or metal-based alloys – in the latter case, the technique is called selective laser melting (SLM)—are used. As locally high temperatures are generated by the laser, materials must be thermally stable up to high temperature (>150 °C), and the encapsulation of cells and biological factors is excluded. Using multiple materials is not trivial with this technique, but a high control can be exerted over the architecture and is thus suited for the implementation of architectural gradients in a scaffold.

## 3 Gradient Scaffolds for OC Regeneration

In the last decade, researchers changed their focus from homogenous to gradient-type scaffolds. AM techniques, especially extrusion-based, were applied to incorporate architectural, compositional, and thereby mechanical gradients in scaffolds. The fate of seeded cells in such scaffolds can be highly different, depending on their location. In this section, we provided an overview of the incorporation of material gradients in AM scaffolds, and their effect on seeded cells in view of OC regeneration. A distinction was made between solid (Section 3.1), hydrogel (Section 3.2), and hybrid scaffolds (Section 3.3). At the end of each sub-section, challenges and recommendations are discussed that may improve OC scaffold design.

### 3.1 Solid AM Scaffolds

Solidified scaffolds are typically composed of synthetic materials that exhibit a melting temperature. These materials are processed at higher temperatures with techniques such as ME-AM, MEW, or SLS, after which they solidify and form a scaffold upon cooling. To mimic the various gradients found in the OC unit, the architecture or composition can be varied across the construct.

#### 3.1.1 Architectural Gradients

When fabricating AM scaffolds for OC regeneration, it is important to consider the architectural gradients, such as the porosity of the subchondral bone, present in the native tissue. AM enables the fabrication of scaffolds containing a gradual change in porosity of each layer.^[62–64]^ In addition to porosity, AM techniques enable the fabrication of scaffolds with pore shape gradients, such as rhomboidal or gyroid-like shapes, which resemble the native bone architecture more closely.^[62,63]^ Alongside these architectural gradients, typically mechanical gradients across the scaffold are introduced. Layers with a higher porosity are easier to deform, lowering the overall mechanical properties of the scaffold.^[65,66]^

Pore size, pore shape, and porosity, as well as the typically associated architectural gradients influence cell differentiation and have led to heterogeneous tissue deposition in constructs. For example, Di Luca and coworkers fabricated an AM scaffold with a stepwise decrease in porosity from ≈80% to ≈55%. Human mesenchymal stromal/stem cells (hMSCs) were seeded in these gradient scaffolds. Evaluation of the gene expression in chondrogenic medium in the different regions of scaffolds revealed that aggrecan, collagen II, and Sox 9 were upregulated when the pore sizes were smaller. A similar trend was observed in the normalized production of GAGs. Notably, in basic medium, the pore size did not affect the production of chondrogenic markers, suggesting a combinatorial effect of media and pore shape. The authors hypothesized that smaller pores contained a higher cell density, and consequently a more hypoxic environment, which stimulated chondrogenic differentiation.^[67]^

Interestingly, Kamboj et al. produced scaffolds with pore size gradients using SLS technology, and reported an increase in expression of osteogenic markers at relatively small pore sizes (<50 μm).^[64]^ In the latter study, wollastonite—a calcium silicate mineral—was used as substrate. Simultaneously, it was suggested that a scaffold with medium-high porosity (65–75%) favors bone regeneration and vascularization in-vivo.^[68]^ The reported opposite findings can most likely be attributed to the type of material used, as the intrinsic properties of the substrate have a strong effect on cell behavior as well.^[69]^ Overall, these studies indicate an optimal architecture exists for a certain biomaterial, which may lead to synergistic effects.

#### 3.1.2 Compositional Gradients

As mentioned in the introduction, compositional gradients are also present in the OC unit. Cells seeded in scaffolds manufactured from different materials change their fate depending on their location in the scaffold. One of the most suited AM techniques to integrate compositional gradients is ME-AM, because it allows to switch cartridges during the fabrication process. Other techniques often do not enable facile switching of reservoirs to create a material gradient. For example, Du and coworkers used SLS to fabricate a construct with a gradient of TCP particles, but manual addition of the TCP was required during scaffold fabrication to change the mineral content.^[70]^

**Figure 3:**
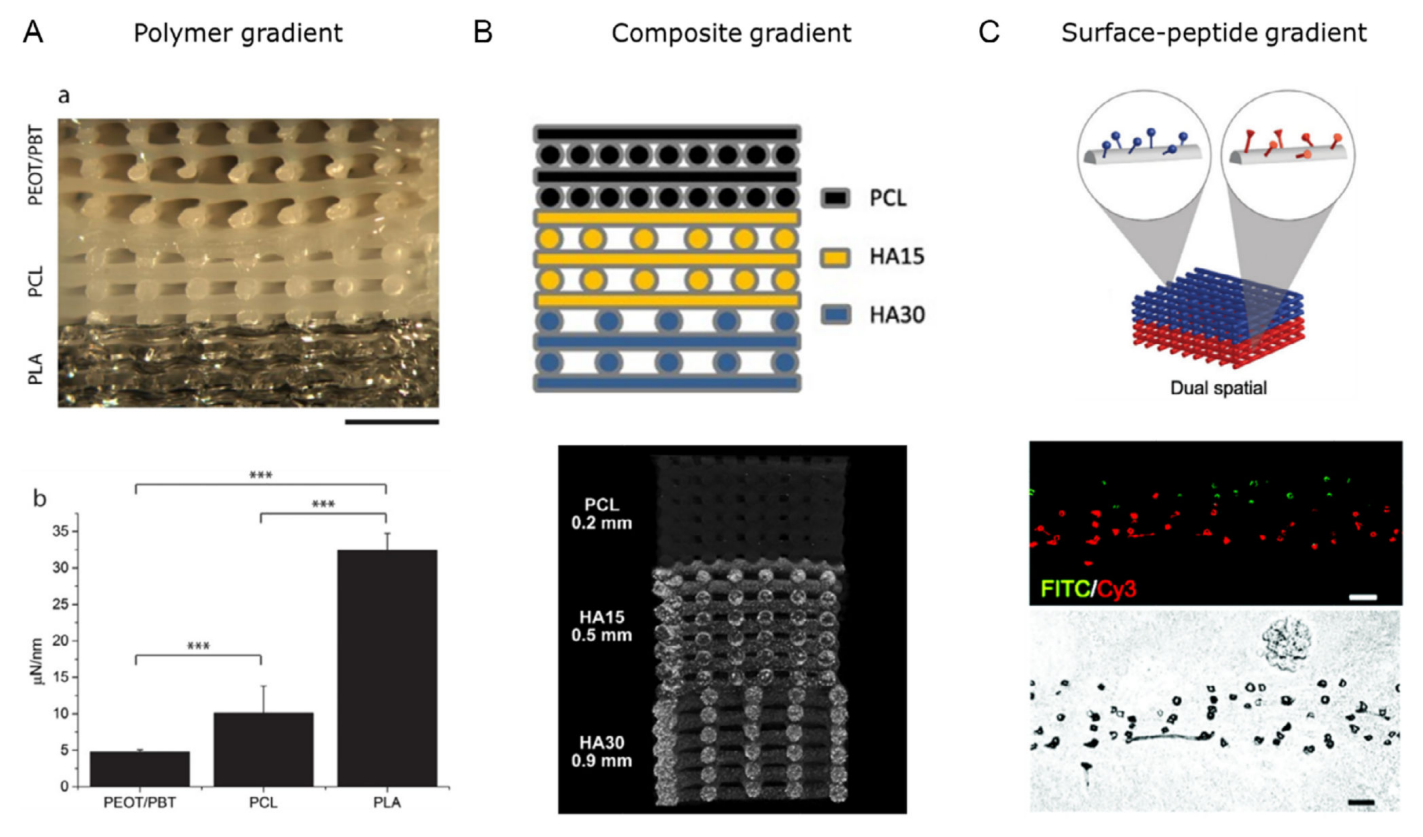
Examples of scaffolds containing compositional gradients produced via ME-AM. A) Di Luca et al. fabricated a tri-layered scaffold comprising PLA, PCL, and PEOT/PBT to provide a compositional and mechanical gradient to seeded cells. Adapted with permission.^[71]^ Copyright 2016, IOP Publishing Ltd. B) Bittner and colleagues manufactured a tri-layered scaffold containing a stepwise increase in HA content as well as a pore size gradient, as shown by the micro CT image. Adapted with permission.^[75]^ Copyright 2019, Elsevier Ltd. C) Chow and coworkers produced a bi-layered scaffold containing chondrogenic and osteogenic differentiation-inducing peptides on opposing sides. The peptides were fluorescently labeled to visualize the gradient. Adapted with permission.^[83]^ Copyright 2021, Royal Society of Chemistry.

Pioneering work in the preparation of compositional gradients using ME-AM was reported by Di Luca and coworkers, who fabricated a tri-layered AM construct by consecutive printing of PLA, PCL, and PEOT/PBT (Figure 3A). They demonstrated that hMSCs residing in the PLA region deposited a high number of GAGs after 28 days of culture in chondrogenic medium, while in the PEOT/PBT region the upregulation alkaline phosphatase (ALP) activity was observed in mineralization medium. Notably, in basic medium, there were no statistical differences found.^[71]^ Given this absent effect in basic medium, it would be interesting to investigate how these results translate in an in-vivo system in future studies. In in vitro studies, optimized differentiation-inducing media can be used to stimulate differentiation of cells, while the environment in vivo cannot easily be tailored. Thus, one could hypothesize that an intrinsic effect of the material is required to control cell differentiation and matrix deposition in vivo.

Instead of only using thermoplastic polymers, titanium-based metals have been added to constructs in the bone domain due to their biocompatibility and excellent load bearing properties (*E* ≈ 1 GPa).^[72]^ For example, a titanium cage-like structure was developed via SLM as support structure for the calcified and articular cartilage domains in a tri-layered construct.^[73,74]^ Here, the cartilage domain comprised of two poly(lactic*-co*-glycolic acid) (PLGA) layers (≈30 μm for the middle layer versus ≈100 μm for the top layer), was deposited on the titanium cage-like construct. Autologous chondrocytes were seeded in the PLGA region, and the full construct implanted in an osteoarthritic goat model for 3 months. As comparison, the defects were also treated with mosaicplasty surgery as well as a similar construct, where the titanium cage was replaced with a TCP one. Macroscopic evaluations of tissue treated with the titanium construct and mosaicplasty surgery revealed the formation of smooth hyalinelike cartilage. Histological evaluations showed the deposition of pre-dominantly collagen II, the formation of a tidemark, as well as the formation of vascularized subchondral bone. In comparison, the use of a TCP-based construct led to the formation of more fibrocartilage-like tissue, indicating a direct effect of the material composition on regeneration. Notably, the addition of a dense PLGA prevented the overgrowth of both bone and cartilage.^[74]^ Due to the absence of an untreated empty defect, the effect of the intrinsic healing capacity of the goat could not be compared.

The drawback of using purely synthetic materials is their lack of biologically relevant moieties, which limits their ability to direct cell fate in a controlled fashion. To this end, composite materials comprising thermoplastic polymers and inorganic minerals have been used in AM to stimulate the formation of bone tissue. Subsequently, compositional gradients with these composites have been created to mimic the mineralization gradient in the OC unit. For example, in the Mikos group scaffolds were manufactured containing a discrete TCP (0–10–20%) or HA gradient (0–15–30%) (Figure 3B). In addition to the compositional gradient, these scaffolds had increasing porosity, aligned with the mineral gradient.^[75–77]^ The amount of β-TCP was strongly correlated with the upregulation of ALP activity in rabbit MSCs as well as the deposition of mineralized matrix, which was independent of the pore size gradient.^[76,77]^

Inorganic materials are typically added to a polymer to promote osteogenesis in the scaffolds. To stimulate chondrogenesis, naturally occurring ECM components of the AC were blended with synthetic polymers. For example, Natarajan and colleagues manufactured a biphasic PCL/PLGA construct blended with chondroitin sulfate, the main GAG in the cartilage phase, and β-TCP in the bone phase. After 28 days of culture in differentiation media, the expression of late-stage differentiation markers was observed in immunofluorescent images compared to control scaffolds without additives. Addition of chondroitin sulfate stimulated the deposition of aggrecan and collagen II, whereas addition of TCP stimulated the deposition of late osteogenesis markers such as osteocalcin and osteonectin.^[78]^

Instead of blending, synthetic polymers can also be chemically modified with biomolecules such as proteins or small peptides to change their bioactivity. However, due to the high temperatures involved in, for example, ME-AM or SLS, most proteins are unable to retain their biological activity, and consequently can only be introduced post-printing. Since peptides typically do not rely on folding mechanisms, these can be coupled to the polymer prior to printing. The most widely used peptides to induce chondrogenesis or osteogenesis are derived from or interact with transforming growth factor (TGF)-β1/3 and bone morphogenic protein (BMP)-2/7, respectively, although many other peptides involved in OC regeneration have been investigated, and reviewed elsewhere.^[79,80]^ By controlling the spatial distribution of peptides, cells can be directed toward different lineages within a scaffold. For example, Guo et al. prepared a PCL polymer with alkyne end groups, which were used to anchor cartilage- (N-cadherin mimetic) and bone-specific (BMP-mimetic and glycine-histidine-lysine) peptide moieties. ME-AM was subsequently used to fabricate a scaffold with a discrete peptide gradient. After extrusion, the peptides retained their bio-activity as a positive effect was observed on the calcification of the matrix as well as GAG deposition of seeded hMSCs, respectively.^[81]^ The Chow group used a slightly different approach by blending peptide-conjugated lower molecular weight PCL with high molecular weight PCL.^[82]^ The advantage of modifying the lower molecular weight polymer in the blends is that the number of moles of peptides per gram increases, which theoretically leads to a higher density on the surface. Camacho et al. used two cartridges with PCL-based blends, containing either a conjugated HA-binding peptide or an E3 (mineralization) peptide, to fabricate a bi-phasic construct (Figure 3C). The scaffolds presenting both peptides in opposing zones promoted upregulation of early chondro- (i.e. Sox9) and osteogenic (i.e. RUNX2) differentiation markers of hMSCs simultaneously after 14 days of culture in basic medium. After 28 days of culture immunofluorescent imaging showed the deposition of collagen II and X in the “chondrogenic” and “osteogenic” domain of the scaffolds, respectively.^[83]^ Notably, collagen I was deposited throughout the full scaffolds, indicating the formation of hypertrophic chondrogenic or osteogenic phenotypes. These studies highlight the importance of controlling the spatial organization of biochemical cues to direct the formation of heterogenous tissue in OC scaffolds.

#### 3.1.3 Post-Modification of an AM Surface in Gradient Fashion

Surface modification strategies post-printing can be used to modify the biological properties of a scaffold. A gradient of surface peptides was shown to gradually direct hMSCs differentiation toward chondrogenic, osteogenic, and mixed lineages in 2D.^[84,85]^ Although there are several surface modification strategies available for AM scaffolds, both non-covalent and covalent, the introduction of the bioactivity in gradient fashion remains challenging. For example, aminolysis reactions or plasma treatments are common approaches to form amine or carboxylic acid groups, respectively, on the surface of polyester-based scaffolds.^[86,87]^ These reactive groups can subsequently be used to introduce bioactive molecules such as differentiation-inducing peptides^[88]^ or proteins.^[89,90]^ However, these approaches typically target the scaffold homogeneously.

**Figure 4:**
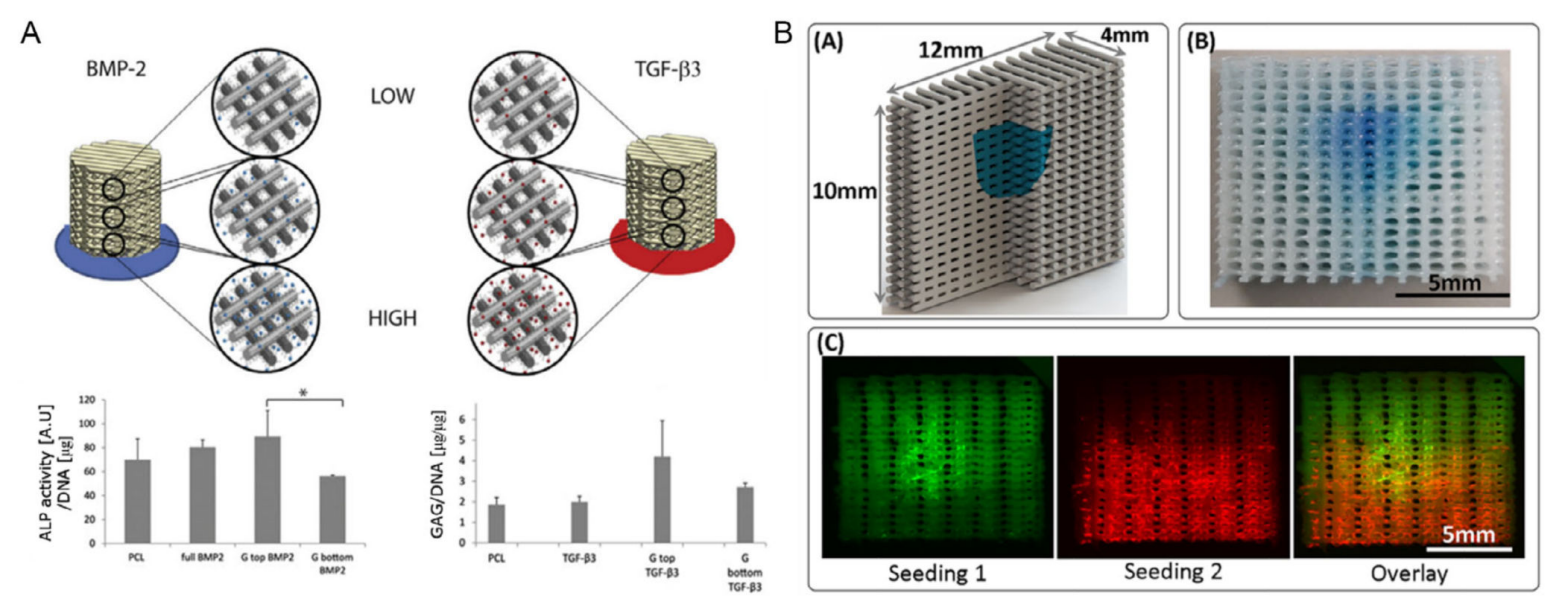
Incorporation of bioactive groups in gradient fashion via modification strategies post-printing. A) Di Luca and coworkers used capillary forces to attach BMP-2 or TGF-β3 in gradient fashion on the surface via carbodiimide chemistry and studied its subsequent effect on hMSC differentiation. Adapted under the terms of the CC-BY 4.0 license.^[91]^ Copyright 2017, The Authors, published by Wiley. B) Sinha et al. used an integrated plasma torch to modify the surface of AM fibers in a spatially controlled manner. The patterning enabled region-selective attachment of cells (seeding 1). Reproduced under the terms of the CC-BY 4.0 license.^[94]^ Copyright 2021, The Authors, published by Springer Nature.

To circumvent a uniform distribution of the bioactive component by post-modification of the scaffold, Di Luca and coworkers used capillary forces to react proteins (TGF-β and BMP-2) in gradient fashion across a scaffold. Toward the higher concentration of the proteins an enhanced GAG production and ALP activity, respectively, was observed in hMSCs, after 28 days of culture in basic medium (Figure 4A).^[91,92]^ Another strategy was shown by D’Amora and coworkers, who used a dip-coater to slowly submerge a scaffold in solution causing the introduction of a gradient of amine groups via aminolysis. Subsequently, this gradient was used to graft collagen I on the surface applying EDC/NHS chemistry.^[93]^ To achieve a heterogenous distribution of reactive groups via plasma treatment, novel AM technology were developed. For example, Sinha et al. developed a hybrid AM platform with an integrated plasma jet, which enabled the local treatment of the scaffold with plasma. This technique created a localized cell adhesive region in the scaffold (Figure 4B).^[94]^ This concept enables the seeding of different cell types, such as chondrocytes or osteoblastic cells, in different regions of the scaffold, which may promote localized tissue production.

#### 3.1.4 Recommendations

Although the OC is generally depicted as a zonal tissue, the tissue does not contain highly defined interfaces, with exception of the onset of mineralization at the tidemark. Scaffolds that contain discrete gradients often have interfaces that are prone to delamination upon applied shear forces. However, the implementation of gradual transitions in AM scaffolds is challenging for some of the techniques described earlier.

From an architectural point of view, continuous gradients have been successfully introduced using ME-AM, DLP, and SLS. DLP and SLS especially enable the fabrication of complex architectures. ME-AM scaffolds are often designed with a woodpile architecture, which does not mimic the native tissue architecture. Recently, ME-AM was used to fabricate hypotrochoidal scaffolds, resembling the collagen orientation of the cartilage.^[95]^ Another interesting way forward could be the fabrication of unconventionally shaped scaffolds using a robotic arm.^[96]^

ME-AM enables the use of multiple cartridges, but it generates discrete scaffolds with interfaces. Recently, a novel AM print head containing two reservoirs was designed that enable the gradual mixing of (inorganic) materials and polymers in the extrusion channel.^[94]^ In future research, this print head could be used to create a variety of compositional gradients in a scaffold. Here, a careful selection is required to ensure the miscibility of polymers to prevent phase separation.^[97]^ For OC applications, we recommend investigating the integration of a poly(ester urethane)s material into a gradient construct. Camarero-Espinosa et al. used this polymer to fabricate AM scaffolds with much higher resilience (yield strain = ≈30%) than typical polymeric scaffolds such as PLA (yield strain = ≈5%).^[26]^ We believe that a combination of such a poly(ester urethane) and a composite could be an interesting future avenue to explore for the integration of cartilage- and bone-like properties into an AM scaffold.

As previously discussed, the drawback of current postmodification methods is their inability to create gradients across a construct. One reason is the nonspecific reaction between the liberated reactive group and targeted molecule. To increase the specificity as well as efficiency of the surface reaction, a solution could be the introduction of bio-orthogonal groups allowing click reactions on the fiber surface. Typical examples of these “click” reactions are copper(I)-catalyzed or strain promoted azide–alkyne cycloaddition, photo-initiated thiol-ene coupling, (inverse demand) Diels–Alder reaction, and oxime ligation.^[98–100]^ The Becker group demonstrated that osteogenic-inducing peptides (BMP-2 and BMP-7 derived) could be grafted on the surface via alkyne-azide cycloaddition chemistry. To this end, a novel poly(ester urea) polymer was synthesized, in which part of the backbone contained a pendent alkyne group.^[101]^ The introduction of this reactive group in gradient fashion could be leveraged to create a surface gradient.

### 3.2 Hydrogel AM Scaffolds

The nature of solid scaffolds prepared from thermoplastic polymers does not resemble the water-retaining properties of articular cartilage, which is a highly hydrated tissue. Articular cartilage possesses stress-relaxing and resilient properties, enabling it to dissipate energy under load.^[102]^ These properties tend to be limitedly present in thermoplastic synthetic polymers, but are typically observed in hydrogels. Hydrogels that resemble the composition of the cartilage matrix tend to favor upregulation of Sox9 in chondrocytes and MSCs.^[103,104]^ Sox9 is considered the master transcriptional regulator for chondrogenesis.^[105,106]^

#### 3.2.1 Compositional Gradients

Hydrogels do not resemble the composition of the bone matrix. To fabricate a hydrogel scaffold for OC applications, gradients of inorganic materials were introduced such as ones of (nano) hydroxyapatite (nHA),^[107–110]^ TCP,^[111]^ or bioactive glass.^[112]^ These multi-layered hydrogels are typically manufactured via consecutive extrusion of (bio)inks. These constructs are often stabilized via post-UV crosslinking. For example, Zhang and coworkers fabricated a nHA gradient in a bone marrow derived MSC (BM-MSC) laden alginate tri-layered scaffold (0–40–70% w/w). The construct induced neo-cartilage formation (Collagen II ↑) as well as allowed for bone integration in an osteoarthritic rat model.^[110]^

**Figure 5:**
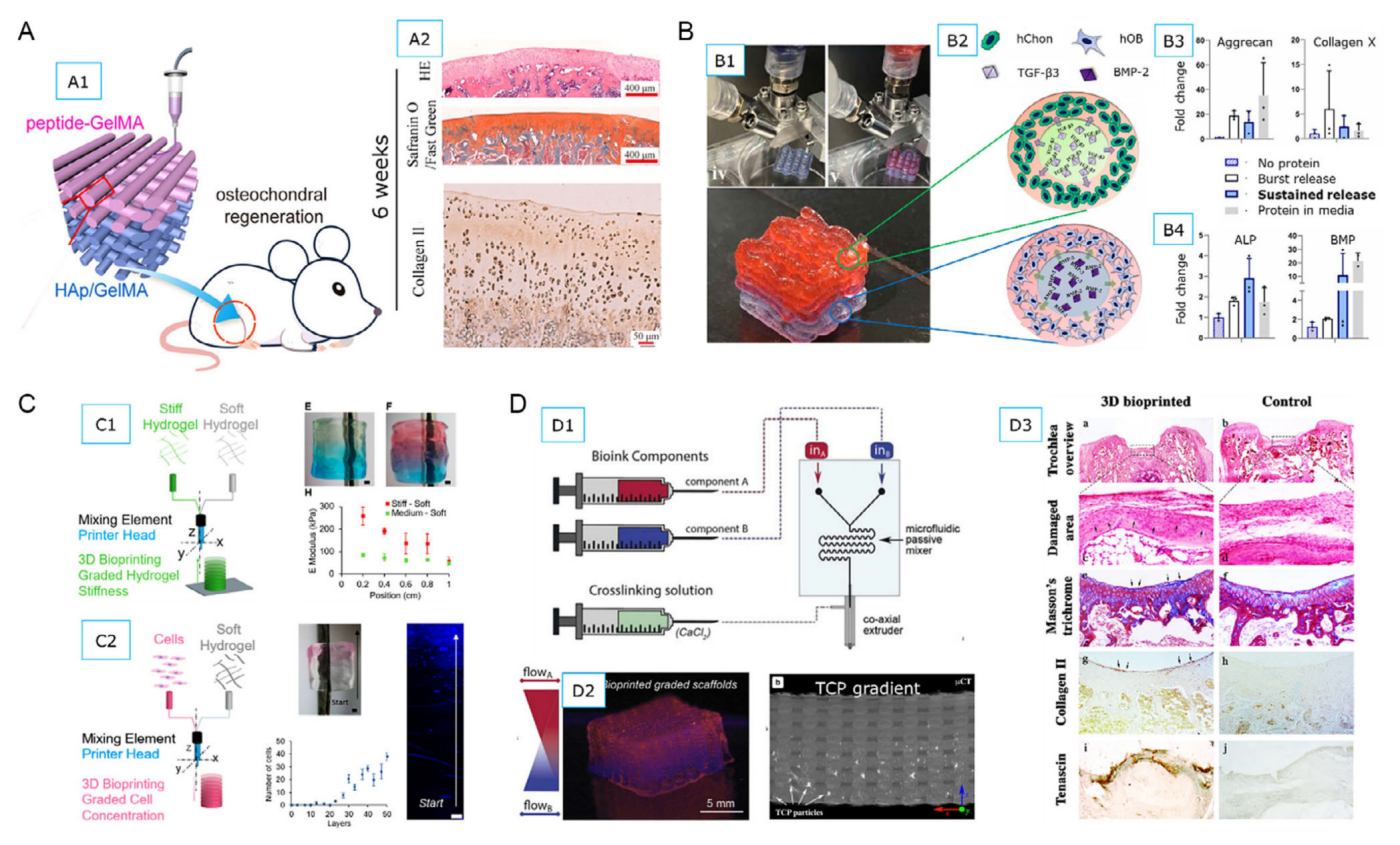
Examples of hydrogel scaffolds comprising gradients for OC applications. A) In the Ding group, a bi-layered GelMA-based hydrogel was fabricated via extrusion-based AM; the cartilage phase was supplemented with a TGF-β1 binding peptide and the bone phase with HA (see A1). The construct was stabilized by UV crosslinking post-printing. After 6 weeks of implantation in an osteoarthritic rat model, the newly formed tissue was evaluated via histology, such as H&E, Safranin-O, and collagen II staining, indicating the presence of hyaline-like cartilage (see A2). Adapted with permission.^[108]^ Copyright 2022, American Chemical Society. B) Core–shell fibers were deposited in a bi-layered fashion using a conical-shaped coaxial nozzle (see B1). The core contained (charged) laponite that enabled the sustained release of bioactive factors (i.e. TGF-β3 and BMP-2) to cells (i.e., either human chondrocytes or osteoblast, respectively) in the shell (see B2). The localized delivery of proteins via sustained release stimulated chondrogenic (see B3) and osteogenic (see B4) genes in the immediate adjacent cellular environment, while undesired offsite effects on the cells in the opposing compartment remained absent. Adapted under the terms of the CC-BY 4.0 license.^[114]^ Copyright 2022, The Authors, published by IOP Publishing Ltd. C) Kuzucu et al. used a custom-made mixing head prior to deposit hydrogels scaffolds comprising a continuous stiffness (see C1), peptide, or cellular gradient (see C2). Adapted under the terms of the CC-BY 4.0 license.^[118]^ Copyright 2021, The Authors, published by American Chemical Society. D) Idaszek et al. designed a microfluidic print head to control the inflow rate of two materials. Real-time control over the mixing was used to create a continuous TCP gradient in a construct. After 12 weeks of implantation in an osteoarthritic rat model, IHC results (i.e., H&E, Masson’s trichrome, collagen II and tenascin) were compared to an empty defect control, indicating the presence of cartilage-like tissue (see D2 & D3). Adapted with permission.^[120]^ Copyright 2019, IOP Publishing Ltd.

Besides supplementing mineralized components to the bone region of the hydrogel, chondrogenesis promoting factors such as TGF- β,^[111]^ manganese ions,^[112]^ or kartogenin^[113]^ have been added to the cartilage mimetic region. For example, the Ding group used extrusion-based printing of GelMA and GelMA reinforced with nHA, followed by UV crosslinking.^[107,108]^ In one of their studies a bi-layered hydrogel, with a TGF-β1 binding peptide incorporated in the cartilage domain, was implanted in an osteoarthritic rat model (Figure 5A). After 6 weeks, this construct supported the formation of hyaline-like cartilage, as indicated by the strong safranin-O staining for GAGs. In addition, collagen II was abundantly present, except for the superficial layer. The HA-loaded region of the scaffold permitted bone ingrowth, as well as increased bone regeneration (BV/TV ↑).^[108]^ Gao et al. manufactured a bi-layered hydrogel comprising manganese ions and bioactive glass in the cartilage and bone layer, respectively. In this study, GelMA was modified with poly(*N*-acryloyl 2-glycine) groups, which form self-complementary hydrogen bonds, to improve the shear thinning and mechanical properties (tensile strength went up to 1.1 MPa). The hydrogel significantly improved OC regeneration in a rabbit compared to an empty defect. After 12 weeks, well-integrated cartilage, with similar thickness than the adjacent cartilage, and characteristic markers was formed (collagen II ↑, GAGs ↑, collagen I ↓). The bioactive glass had a positive effect on the filling of the defect area and subsequent formation of subchondral bone (collagen I, osteocalcin, and BV/TV ↑).^[112]^ In a more recent study, Killian et al. used a multi-channel printer to fabricate a bi-layered hydrogel consisting of core-shell fibers to enable the controlled delivery of growth factors to cells. The shell comprised a hydrogel (3 wt% alginate and methylcellulose) loaded with human chondrocytes or osteoblasts. Laponite nanoparticles (≥3 wt%) were added to the cellfree core to bind either TGF-β3 or BMP-2 for their sustained delivery, respectively, to the shell (Figure 5B). They assessed the effect of proteins on the cells in the opposing compartment with qPCR evaluations. On one hand, no suppression of chondrogenic markers (e.g., aggrecan and collagen II) was observed in chondrocytes when BMP-2 was released in a sustained manner, while on the other hand osteogenic markers (i.e., collagen X, collagen I, and ALP) were upregulated in osteoblasts. Similarly, sustained release of TGF-β3 positively affected chondrocyte markers (e.g., aggrecan and collagen II) and did not suppress osteoblastic markers (e.g., osteocalcin, ALP, BMP, and collagen I).^[114]^ The study highlighted that controlling the release of growth factor is important to prevent undesired differentiation in off-target areas of the scaffold. Yet, performing a similar study with hMSCs would be interesting in the authors’ opinion due to their potential to differentiate towards both chondro- and osteogenic cell types.

Except for 3D-plotting technologies, other techniques were also used for gradient scaffolds fabrication. For example, Shopperly et al. used DLP to manufacture a cartilage-like (curved) construct from multiple resin baths containing porcine chondrocytes. A custom-made build plate enabled switching between different resin baths, composed of weight ratios of GelMA versus HA methacrylate, to introduce a compositional and mechanical gradient simultaneously. Immunohistochemical (IHC) staining revealed an enhanced collagen II and GAG deposition in the superficial areas of the scaffolds that was comprised of higher amounts of gelatin. Notably, higher amounts of gelatin appeared to upregulate gene expression of collagen I simultaneously, yet the subsequent translation into ECM deposition was not verified by IHC.^[115]^ In another study, aspiration-based AM was used to deposit a hydrogel in the voids of a ME-AM pin array. On top of the vertically aligned pillars, an ADSC laden alginate hydrogel was merged in a perpendicular orientation, creating a mechanical gradient. The overall compressive modulus of the construct (1.09 ± 0.08 MPa) was in range with human articular cartilage. Aligning the fibers in a specific orientation assisted the organization of pre-differentiated ADSCs (into chondrocytes) as well as of collagen within 7 days of culture.^[116]^

Due to the aqueous nature of hydrogels, the miscibility of hydrogel precursors is typically higher than thermoplastic polymers, which prevents the formation of distinct interfaces. Therefore, hydrogel constructs for OC applications were designed containing continuous gradients. For example, the Khademhosseini group developed an AM platform that enables extrusion of up to 7 hydrogels simultaneously. A valve system controls the in-flow of the seven reservoirs into the nozzle.

The system was able to pattern cells as well as deposit nHA gradients, indicating its potential for OC applications.^[117]^ Kuzucu and coworkers used a custom-made mixing head to controllably blend two polymers, enabling the fabrication of continuous gradients of stiffness, peptide, and cellular gradients (Figure 5C).^[118]^ This latter technology could be used to create a gradient in the cell density across a construct, as seen in hyaline cartilage, while controlling differentiation via concomitant compositional and mechanical gradients. Furthermore, microfluidic print heads enable the fabrication of continuous gradients by pre-mixing hydrogel precursors in a controlled fashion.^[119,120]^ For example, Idaszek et al. used a flow ramp to co-extrude two bioinks in a continuous fashion. A gradient scaffold comprising a hMSC/chondrocyte-laden “hyaline cartilage (HC)” (a mixture of alginate, GelMA, and CSMA) and a hMSC-laden “calcified cartilage (CC)” bioink (the HC bioink, supplemented with HA methacrylate and TCP) was fabricated (Figure 5D). The HC and CC formulations promoted in vitro the deposition of collagen II, and collagen II as well as *X*, respectively. The gradient construct was implanted in an osteoarthritic rat model for 12 weeks. This implant supported the regeneration of articular cartilage as indicated by the presence of collagen II and tenascin, which remained absent in an empty defect (Figure 3 and 5D). The formation of calcified tissue was not assessed in-vivo.^[120]^ Finally, recent advances in the DLP technology enabled the fabrication of multimaterial scaffolds containing a continuous soft-stiff gradient. Ge and coworkers fabricated a meniscus-shaped scaffold with a stiffness range from 0.6 to 5 MPa along the *z*-axis of the scaffold.^[121]^

#### 3.2.2 Recommendations

Many studies that applied gradient hydrogels for OC regeneration focused on the introduction of a mineralized component to mimic a feature of the bone matrix. However, there are other compositional gradients present, which contribute to characteristic functions of the OC unit. For example, toward the deep zone in cartilage the abundance of negatively charged GAGs such as keratan and chondroitin sulfate increases. The charged GAGs fulfill an important role under compression due to their ability to redistribute water.^[122]^ This function arises from their bottle brush like organization, which creates a positive swelling pressure. Moreover, these GAGs fulfill an important role in tissue homeostasis.

The integration of hydrogel precursors that mimic the properties of GAGs in gradient fashion may be beneficial for cartilage applications. For example, the introduction of sulfate groups into a hydrogel enables the sequestering of a wide variety of positively charged proteins,^[123]^ like chondrogenic-inducing TGF-β.^[34]^ Another strategy was followed by Xie et al. who demonstrated that a bottlebrush polymer, comprising a HA backbone and sulfonated polymeric side arms, strongly interacted with collagen II. Moreover, this polymer provided a very low friction area, which resembles an important characteristic of the AC.^[124]^ Similarly, other synthetic bottlebrush polymers with charged side arms demonstrated the formation of a surface with a very low friction coefficient.^[125,126]^ Thus, the integration of bottlebrush polymers with charged side arms appears beneficial to enhance the biomimetic properties of the hydrogel component.

### 3.3 Hybrid Scaffolds

We have focused in this review on the fabrication of solid or hydrogel scaffolds containing gradients. In this section, we provide an overview of hybrid scaffolds, and their effect on OC regeneration in vitro and in vivo.

#### 3.3.1 Hydrogel Reinforcement via a Supporting Framework

Hydrogels, both from synthetic and natural origin, often do not possess sufficient mechanical properties to withstand the high loads exerted in the OC unit. Therefore, several strategies have been applied to increase the strength of hydrogels such as the formation of an interpenetrating network^[127]^ or composites.^[128]^ In this review, we highlight how AM techniques have been used to strengthen hydrogels by providing a thermoplastic support framework.

The Kelly group showed that the addition of a woodpileshaped ME-AM framework to an alginate hydrogel enhanced the compressive modulus from ≈0.01 MPa to above 0.1 MPa.^[129]^ Moreover, they demonstrated that the geometry of a thermoplastic framework enabled tailoring of the mechanical properties toward native cartilage ECM.^[130]^ In addition to ME-AM frameworks, MEW has been used for hydrogel reinforcement.^[131,132]^ For example, Burdick and coworkers showed that the addition of PCL microfibers enhanced the compressive modulus of norbornene substituted HA hydrogels ≈50 times. Importantly, the addition of the framework did not change the chondrogenic potential of the HA as indicated by the similar collagen II and GAG production after 56 days.^[133]^

Next to fulfilling a reinforcement, the framework also provides a protection function to hosted cells during the initial phase after implantation. In this case, the cell-laden hydrogel is deposited within the voids of the scaffold’s architecture.^[134]^ For example, a PCL-alginate construct containing encapsulated chondrocytes was implanted in a nude mice model subcutaneously for 4 weeks. The framework provided a cell-friendly ectopic environment to enable the deposition of cartilage tissue (collagen II ↑, GAGs ↑).^[135]^ As slower degrading polymers may eventually hinder tissue ingrowth, a faster degrading polymer could be selected. A PLGA framework with a hMSC-laden hydrogel was completely absorbed after a 24-week implantation period in an osteoarthritic rabbit model. The hMSCs within the framework were able to deposit full-thickness hyaline-like cartilage, as indicated by the GAG formation and cartilage-like collagen orientation observed via IHC, and bone formation (≈40% BV/TV), as shown by microCT analysis. The control samples (i.e., hydrogel only and reinforced hydrogel without cells) displayed thin fibrocartilage-like tissue, likely as result of hydrogel loss and limited infiltration of cells, respectively.^[136]^ These results suggest that the addition of a protective framework facilitates the homogenous distribution of cells over time as well as it provides time to deposit new tissue across the full defect thickness.

Different types of gradients can be introduced in these hybrid scaffolds. For example, in multi-layered hybrid scaffolds, the architecture of the framework can be varied, and leveraged to introduce a mechanical gradient across the construct.^[137,138]^ In addition, when both hydrogel and solid frameworks are deposited via AM techniques, different bioinks were patterned alongside the framework, creating a cellular gradient.^[139]^ Furthermore, surface modifications post-printing have been used to create a compositional gradient of bioactive molecules, which were used to direct MSC spheroid maturation toward specific OC lineages in a spatially controlled manner.^[140,141]^ For example, Lee and coworkers used polydopamine coatings to anchor TGF-β3 and BMP-2 on PCL frameworks. These scaffolds permitted in an osteoarthritic rabbit model the regeneration of hyaline cartilage formation (20% complete, and 60% predominantly hyaline according to histological scoring) and the formation of subchondral bone (≈80% comprised of trabecular bone).^[141]^

#### 3.3.2 Multi-Layered Hybrid Constructs

Most multi-layered hybrid scaffolds applied for OC regeneration consist of three zones: cartilage, calcified cartilage, and the subchondral bone. The hydrogel applied to represent the cartilage phase is often reinforced with a MEW or ME-AM framework. Dispersion of the hydrogels throughout the framework can be done via molds or extrusion alongside the solid fibers. The extrusion of hydrogels resembling the (calcified) cartilage phase is mostly done via 3D plotting, but in some cases DLP was used.^[142,143]^

**Figure 6:**
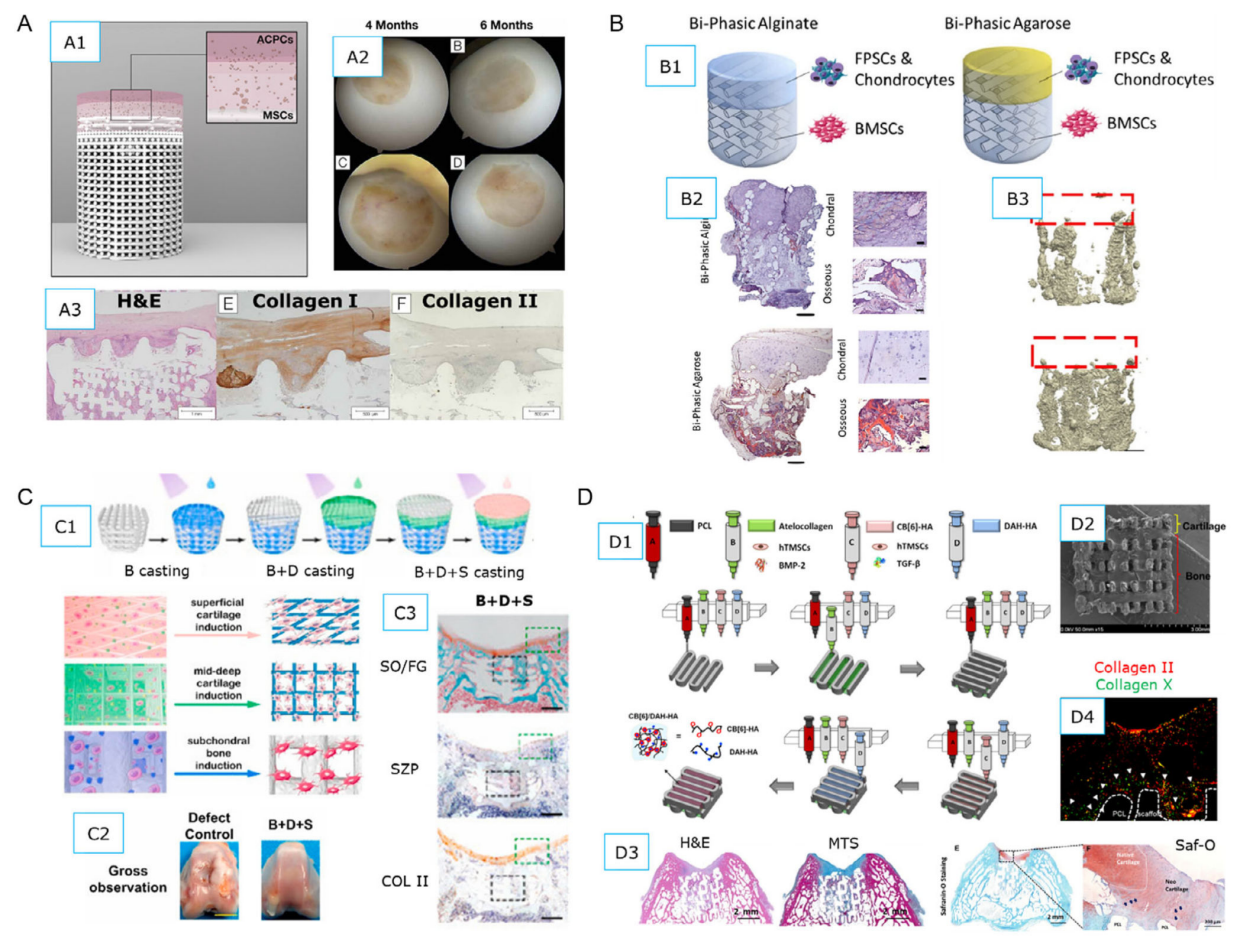
Fabrication of hybrid constructs comprising gradients for OC applications. A) Mancini and coworkers developed a tri-layered construct with a partially reinforced bi-layered cell-laden hydrogel (see A1). The PCL framework contained a pore size gradient resembling the architectural gradient found across the transition from cortical to trabecular bone. After 6 months of implantation in an equine knee, arthroscopic and histology revealed the presence of fibrocartilage (See A2 and A3). Adapted with permission.^[144]^ Copyright 2020, IOP Publishing Ltd. B) Bi-layered reinforced hydrogels that contain encapsulated cells can be dispersed through a uniform thermoplastic framework, prepared via ME-AM (see B1). MicroCT analysis indicated that the biphasic construct prevented overgrowth of the bone (see B2 and B3). Adapted under the terms of the CC-BY 4.0 license.^[147]^ Copyright 2020, The Authors, published by Elsevier Ltd. C) Qiao and coworkers casted (rabbit) BMSC-laden hydrogels throughout a solid tri-layered framework produced by MEW as well as ME-AM. The architecture of the framework was inspired by the architectural gradient of the collagen orientation across the OC unit (see C1). The hydrogels were loaded with TGF-β1 (hyaline cartilage), BMP-2 (subchondral bone), or both growth factors (calcified cartilage) to assist zonalspecific differentiation in each layer. Implantation for 24 weeks led to the formation of neo-cartilage with a smooth appearance (see C2) and the OC unit contained zone-specific markers such as GAGs, collagen II and collagen I. Adapted with permission.^[148]^ Copyright 2021, Elsevier Ltd. D) Instead of dispersion, Shim and coworkers used extrusion-based AM to deposit a gradient of cell-laden hydrogels, loaded with either chondrogenesis or osteogenesis promoting factors, alongside an ME-AM reinforced framework (see D1 and D2). The construct with a dynamic hydrogel permitted the formation of GAG and collagen II rich cartilage, as well as bone formation (see D3 and D4). Adapted with permission.^[152]^ Copyright 2016, IOP Publishing Ltd.

Mancini and coworkers developed a PCL-based tri-layered scaffold that partially reinforces a double layered cell-laden HA/poly(glycidol) hydrogel. The PCL framework contained a pore size gradient resembling the architectural gradient of the cortical to trabecular bone. The unenforced part of the hydrogel contained encapsulated articular cartilage progenitor cells (ACPCs), whereas the other layers contained MSCs (Figure 6A). ACPCs do not express RUNX2 and do not tend to differentiate toward hypertrophic chondrocytes. After implantation of the construct in an equine model for 6 months, the osteogenic phase supported the regeneration of the subchondral bone. Notably, the quality of neo-cartilage formed in the scaffold was low (i.e., high amount of collagen I, and scarce expression of collagen II) (Figure 6A3). They attributed the absent effect of the construct to the combination of loss of cells upon implantation, quick hydrogel degradation, and mismatch in biomechanical properties of the hydrogel.^[144,145]^ In their most recent work, the full hydrogel was stabilized in a MEW support framework that would aim to mimick the native collagen orientation, which protected the cells and hydrogel after implantation. Although the performance of the scaffolds was not compared to an empty defect, they observed that constructs with pre-formed cartilage cells and tissue did not outperform a cell-free control in terms of biomechanical and matrix deposition. This result challenges the idea that a construct must be pre-seeded with cells, and that mimicking the architectural gradient of collagen is pivotal for especially cartilage regeneration.^[146]^ The results of these two studies highlight that the complexity of regenerative processes in vivo remains hard to predict, even when a scaffold is rationally designed.

The Kelly group manufactured AM frameworks of PCL, PLA, and PLGA that are fully reinforced with a dispersed bi-phasic hydrogel. The subchondral bone phase contained BM-MSCs and the cartilage phase a co-culture of fat pad derived stromal cells (FPSCs) and chondrocytes, encapsulated in an alginate or agarose hydrogel (Figure 6B). These constructs contained cellular, compositional, and mechanical gradients. After 5 weeks of chondrogenic priming, the constructs were implanted subcutaneously in a mouse for 6 weeks. The chondral region of the scaffold contained cartilage-like tissue, as indicated by positive staining for GAGs and collagen II. Moreover, microCT analysis revealed mineral deposition in the bone region while negligible deposition was observed in the chondral region.^[147]^ This study highlighted that the different phases were required to prevent undesired mineralization in the cartilage phase.

Qiao and coworkers developed a tri-layered construct using both MEW and ME-AM to deposit a support frame for GelMA hydrogels. The framework of PCL-*b*-PEG-*b*-PCL contained an architectural gradient; each zone contained a different orientation, inspired by the natural collagen organization in the OC unit, to assist alignment of cells in-vitro. Immediately after the deposition of each zone, a rabbit BMSCs-laden GelMA solution was dispersed using a mold and crosslinked by UV (Figure 6C). The GelMA contained zonal-specific differentiation factors, creating a protein gradient. The hybrid constructs were implanted in a rabbit OC defect model for 24 weeks. The constructs (“B + D +S”) stimulated the formation of full-thickness well-integrated hyaline cartilage, with a high ICRS score (above 10, indicating nearly normal AC). Histological analysis revealed the deposition of zonal-specific ECM markers, such as collagen II and GAGs in the chondral region, and collagen I in the subchondral bone region. Notably, this study showed the presence of proteoglycan-4, which is pivotal for joint lubrication, in the superficial zone.^[148]^

The (cell-laden) hydrogels can also be extruded on top of or in-between the fibers using a multi-cartridge manufacturing system.^[149–151]^ Kilian and colleagues fabricated a tri-layered construct comprising a compositional gradient of a chondrocyteladen alginate-methylcellulose hydrogel, a reinforced alginate hydrogel with calcium phosphate cement (CPC), and a pristine CPC phase. The alginate-methylcellulose gel supported chondrogenesis in-vitro, but CPC affected the chondrocyte viability.^[150]^ The alginate-methylcellulose hydrogel resembling the cartilage (stiffness ≈35 kPa) was not reinforced, and likely requires full reinforcement for invivo applications. In another approach, Sun and coworkers described the fabrication of a fully reinforced MSC-laden multi-layered hydrogel. PLGA microparticles containing TGF-β3 and BMP-4 were added in each hydrogel to stimulate chondrogenesis and osteogenesis, respectively, in a zonal fashion. As seen in multiple aforementioned studies, the framework protected the encapsulated cells after implantation in an OC defect. The new cartilage tissue displayed zonespecific markers such as aggrecan, collagen II, proteoglycan-4 in the superficial area. In the subchondral bone, vessel ingrowth as well bone formation was observed.^[151]^

Similarly, Shim et al. fabricated a construct in which two hydrogels are deposited alongside a PCL framework in bi-phasic fashion (Figure 6D). During the printing process, first a human turbinate-derived mesenchymal stromal cells (hTMSC)-laden collagen bioink containing microspheres loaded with BMP-2 was deposited in the bone phase, and subsequently a HA supramolecular bioink containing microspheres loaded with TGF-β in the cartilage phase. After implantation in the knee of an osteoarthritic rabbit model, the constructs with encapsulated TMSCs in a collagen or supramolecular hydrogel stimulated bone and neocartilage formation, respectively. Notably, in the cartilage phase a dynamic bioink, based on host-guest chemistry was used. The effect on cartilage regeneration of this dynamic bioink was compared to an alginate-based bioink in another part of the study. The dynamic hydrogel had a higher expression of collagen II and X on the superficial and around the calcified cartilage area, respectively. Moreover, there appeared to be better tissue integration, as indicated by safranin-O staining.^[152]^ This result may be explained by the dynamic behavior of the HA-based hydrogel that resembles the ECM properties of the AC more closely, and highlights that using dynamic materials for OC application could be explored more extensively.

#### 3.3.3 Recommendations and Future Directions

In this review, an overview of the state-of-the-art of gradient scaffold development was provided in view of OC regeneration. We focused on the most used existing AM technologies. AM technology is advancing at a fast pace and novel high technological solutions are being developed to create gradient scaffolds.^[153,154]^ Overall, based on the biological outcomes, the regeneration of full-thickness osteochondral tissues appears to be favored when a synthetic framework is added to a cell-laden hydrogel in the cartilage domain, which reinforces the mechanical properties and protects the cells after implantation. Moreover, a clear distinctive transition between the cartilage and bone phase prevents the overgrowth of the different tissues. Furthermore, the addition of inorganic materials in a porous bone compartment is beneficial for subchondral bone formation.

However, in the gradient scaffolds prepared by current AM techniques some challenges to further advance OC scaffold design can still be identified. Firstly, the chemical integration between the hydrogel and solid framework has not been extensively studied, which avoids delamination between the phases. Moreover, a covalent interaction may further assist dissipation of energy into the framework under compression to approach human values more closely. Secondly, methodological improvements are required to validate the success of gradient constructs in vitro and in vivo, especially in ones with continuous gradients. In many studies, the full construct is investigated with biochemical assays without distinguishing between the different domains. So far, biological stainings are typically used to showcase zone-specific information on tissue formation. Yet, there are large differences in the selection of osteochondral markers that are assessed in each study, making direct comparisons not always straightforward. We believe though that improvements could be made in the standardization and automatization of image analysis to illustrate the presence of a biochemical gradient across a construct. Other methods for tissue evaluation like the use of spatial biology via mass spectrometry imaging could provide more insights into the developed tissue in different areas of constructs.^[155]^ Thirdly, the shape of the newly formed cartilage does not always align with the curvature of the defect, even when the quality of the neo-cartilage tissue from a histological perspective looks promising. A gradient hydrogel with different swelling behavior could be used to introduce the desired curvature into a scaffold.^[156]^ Finally, most applied materials in the OC region do not display dynamic behavior as observed in the native ECM. In the upcoming section, we present types of available dynamic materials and hypothesize how their integration in AM scaffolds could advance the regenerative capacity of OC constructs.

## 4 Dynamic Chemistries to Further Improve Biomimicry

Most materials that have been used in OC applications in-vivo displayed static, elastic behavior.^[19]^ However, the ECM consists of a complex mixture of covalent, non-covalent, and dynamic covalent bonds. These reversible bonds can play an important role in contributing to the dynamic properties of the tissue. For example, in cartilage the network of collagen II and proteoglycans is known to reorganize and exhibit stress relaxation via redistribution of forces and water.^[157,158]^ The importance of matrix dynamics has quickly emerged in tissue engineering, especially in chondrocytes. For example, early reports showed that matrix viscoelasticity can regulate the formation of cartilage matrix formation and influence cell phenotype in vitro.^[159,160]^

In the last decade, novel dynamic chemistry has become available that allows tissue engineers to design and mimic these dynamic features of the ECM. Although only a few materials were applied in an OC context, we believe that these dynamic materials could play a role in this tissue. Here, we will highlight promising dynamic materials that were already proven to be compatible with AM technologies. We envision how their dynamicity could advance the biomimicry of OC constructs.

### 4.1 Supramolecular Polymers

Supramolecular polymers (here defined as polymers that form from supramolecular polymerization) have long inspired bioengineers to mimic the self-assembly of biological networks like actin and collagen. Comprised of smaller synthetic units, supramolecular polymers form via directional stacking or polymerization into larger assemblies, which can nicely mimic the hierarchical molecular structure of the native ECM.^[161]^ Popular motifs in the community include small peptides, peptide amphiphiles, ureidopyrimidinons, and benzene tricarboxamides, to name a few.

Supramolecular polymers from peptides and peptide amphiphiles have a long history in tissue engineering, directly using mimics of protein interactions to create materials. For example, classic experiments with peptide amphiphile nanofibers have shown significant steps forward in the improvement of chondrogenesis from in-vitro to a rabbit model.^[162]^ Furthermore, smaller self-assembling peptides showed the ability to support chondrocyte culture, and lead to enhanced cell survival in-vitro.^[163]^ From these landmark studies, peptide based self-assembling systems can be found in a variety of commercial cell culture products like PuraMatrix and BioGelX. Their use in 3D printing for the OC interface remains relatively unexplored, in part due to the typically weak mechanical properties of peptide self-assembled hydrogels. Recent advances in both 3D printing^[164,165]^ and in toughening of the hydrogels^[166–168]^ may give hints as to the way forward toward OC applications.

**Figure 7:**
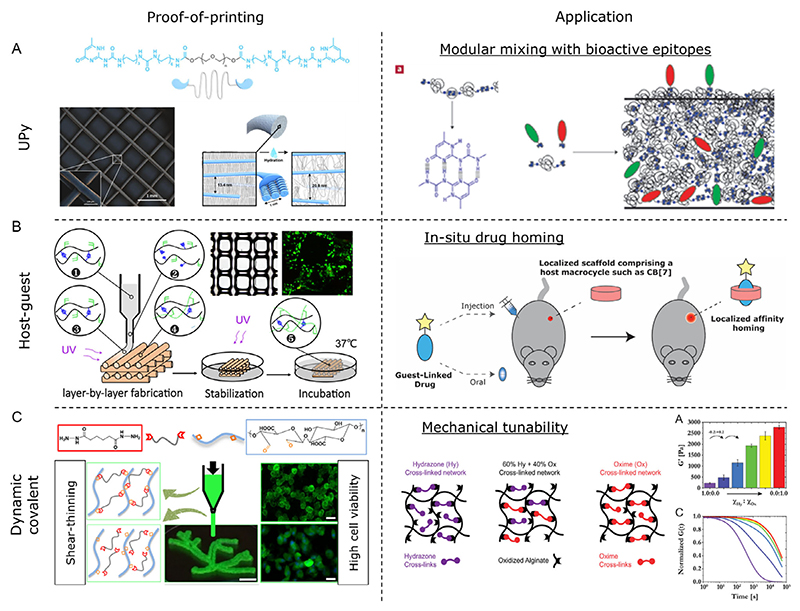
Advances in the development of dynamic materials. In the left panel, their successful utilization in AM applications has been highlighted. In the right panel, a possible exploitation of their dynamicity for OC applications has been depicted. (A, left) Telechelic PEG-diUPy groups were used in MEW to fabricate woodpile scaffolds. Adapted under the terms of the CC-BY 4.0 license.^[169]^ Copyright 2020, The Authors, published by Elsevier Ltd. (A, right) The mix-and-matching ability can be used to introduce relevant bioactive groups on the scaffold. UPy-groups could be modified with chondro- or osteogenic relevant moieties to direct cellular differentiation of seeded cells. Adapted with permission.^[171]^ Copyright 2005, Springer Nature. (B, left) Host–guest chemistry has been used to develop shear-thinning hydrogels, enabling their use in extrusion-based manufacturing. Reproduced with permission.^[189]^ Copyright 2016, American Chemical Society. (B, right) Webber and coworkers have demonstrated that macrocycles such as cucurbiturils displayed a strong enough binding affinity to home drugs in-vivo to the hydrogel location after intravenous administration.^[195]^ This homing ability of hostguest chemistry enables the delivery of growth factors or drugs via injections into the synovial fluid. (C, left) Knowledge over the reversibility of Imine-based dynamic covalent bonds enabled the deposition of shear-thinning bioinks. Adapted under the terms of the CC-BY 4.0 license.^[201]^ Copyright 2018, The Authors, published by MDPI. (C, right) In addition, the difference in molecular constants of various crosslinkers can be used to design mechanically tunable systems with rational control over stiffness and stress–relaxation properties. The tuning of these mechanical properties can be used to direct the fate of encapsulated stem cells. Reproduced under the terms of the CC-BY-NC 3.0 license.^[203]^ Copyright 2022, The Authors, published by Wiley.

Telechelic or chain-extended 2-ureido-4[1 H]-pyrimidinone (UPy) groups, introduced by the Meijer and Dankers groups, form supramolecular biomaterials via dimerization and stacking of self-complementary quadruple hydrogen bonding. These UPy-based supramolecular materials have already been used to fabricate scaffolds with MEW and ME-AM applications (Figure 7A, left).^[169,170]^ The self-recognition through hydrogen bonding enables mixing and matching of biofunctionalized UPy groups such as adhesive domains (UPy-RGD),^[171,172]^ anti-fouling domains (UPy-PEG/UPy-MPC),^[173,174]^ or anti-microbial activity^[175]^ to change the bioactivity of scaffolds (Figure 7A, right). The mixing of UPy-tetrazine into the material enabled functionalization of electrospun fibers via orthogonal inverse electron demand Diels Alder cycloaddition chemistry.^[176]^ From an OC perspective, the mixing and matching ability facilitates the integration of chondrogenic or osteogenic moieties in a scaffold. In addition, UPy groups were modified with atom transfer radical polymerization initiator groups to perform surface-initiated grafting of zwitterionic polymers.^[177]^ The chemical composition of these brushes could be tailored to mimic the negatively charged GAGs chains in the cartilage. These UPy-based solid frameworks could be used to reinforce supramolecular hydrogels, which are typically considered mechanically too weak for AC applications.^[178]^

Another synthetic supramolecular motif that is showing potential in tissue engineering and 3D bioprinting applications is the benzene-1,3-5-tricarboxamide (BTA) molecular unit. BTAs stack via threefold hydrogen bonding around an aromatic core, and have a unique advantage to creating highly dynamic supramolecular polymers in water.^[179,180]^ Only recently have this molecular architectural unit been translated into interacting with cellular units.^[181,182]^ More recently, the molecular architecture of the BTA core to modulate its self-assembly in water has led to the creation of dynamic hydrogels. These hydrogels have shown the ability to modulate the self-assembly of cells,^[183]^ and be amenable to 3D bioprinting via close control over the viscoelastic parameters of the material.^[184,185]^ While most of the BTA hydrogels have viscoelastic properties, they maintain good viability of chondrocytes upon injection and/or culture. Most promising, a recently developed BTA combination material exhibited impressive toughness upon covalent reinforcement of the stack, good processability, and the ability to 3D print a meniscus model, all while allowing hMSC differentiation and early cartilage matrix formation.^[184]^ This newer class of supramolecular materials is especially promising for 3D printing of the OC interface due to their relevant stiffness (≈1 MPa), high toughness, and excellent elastic recovery behavior.

### 4.2 Host-Guest Chemistry

Host-guest chemistry uses macrocyclic hosts that specifically interact with guest molecules through non-covalent interactions. Cyclodextrin (CD) is the most employed macrocycle, and is often known for its function to enhance solubility of drugs.

The Burdick lab used the reversible binding between CD host and adamantane guest to print low viscous solutions in a suspension bath^[186–188]^ or to extrude shear-thinning hydrogels that form fibers upon deposition (Figure 7B, left).^[189,190]^ The binding constant between host and guest depend on the affinity of the guest for the cavity of macrocycles. Host-guest interactions were already used in cartilage applications. β-cyclodextrin and adamantane-functionalized HA enabled sustained release of encapsulated TGF-β1^[191]^ or kartogenin.^[192]^ These “host-guest macromer” hydrogels supported chondrogenesis in-vitro and promoted cartilage regeneration in rats. In addition, due to differences in binding affinity of various guest molecules, hostguest interactions can be leveraged to swap one ligand with another. Pioneering work was presented by Boekhoven and coworkers, who changed the cell adhesive properties of the surface over time.^[193]^ The ability to release or swap upon addition of a competitor molecule could be leveraged to force the release of the bound molecule at a specific moment in time or to change the biological properties of a scaffold. For example, from a cartilage perspective we envision that first a binding epitope is presented to stem cells encapsulated in a hydrogel, followed by a swap for a differentiation-inducing molecule.

The maximum reported binding constant for CD and complementary guests is around relatively low *K*_eq_ (≈10^6^ M^-1^). In-vivo, phenyl groups will compete for occupation of the cavity due to a similar binding constant, which may limit their applicability. In comparison, pumpkin-shaped cucurbituril (CB) macrocycles were reported to interact with a variety of guest molecules spanning a much broader range of *K*_eq_, even up to higher values as observed in-vivo (≈10^15^ M^-1^).^[194]^ The differences in binding affinity of guest molecules for the CB enable the design of mechanically tunable as well as injectable shear-thinning hydrogels.^[194–196]^ However, unlike a CD based system, materials with CB were not yet applied in AM techniques. Notably, the high binding constant of host–guest enabled the homing of intravenously injected drugs into a hydrogel, after its subcutaneous implantation in a rat (Figure 7B, right).^[195]^ Since the hosting of proteins in CB also prolonged their life-time,^[197]^ we believe that this homing ability could be leveraged in OC systems. Biomolecules or drugs with a conjugated guest molecule could be injected into synovial fluid to enable their delivery to the scaffold without being excessively absorbed in the healthy tissue.

### 4.3 Dynamic Covalent Chemistry

More recently, dynamic covalent chemistry was highlighted as versatile tool to design hydrogels. The network of these hydrogels is commonly based on reversible imine-, boronic acid ester-, and Diels Alder chemistry. These dynamic covalent systems are able to break their covalent bond upon a trigger such as pH.^[198–200]^ From a biological point of view, particularly imine-based dynamic covalent chemistry appeared interesting due to its functioning in the physiological pH range. To this end, we will zoom-in on dynamic covalent chemistry.

The molecular equilibrium and kinetic constants (*K*_eq_, *k*_1_, and *k*_-1_) describe the stability, formation, and breakage of these dynamic covalent bonds. Hafeez et al. showed that knowledge of the molecular constants could be used to design printable oxidized alginate based bioinks, which simultaneously maintained good chondrocyte viability (Figure 7C, left).^[201]^ Moreover, these molecular constants enable the design of hydrogels with predictable stiffness, self-healing, and stress relaxation.^[202,203]^ For example, Morgan and coworkers showed that, by creating a mixed system of oxime (high *K*_eq_) and hydrazone (low *K*_eq_) cross-links, both stiffness and stress relaxation properties were tunable (Figure 7C, right).^[201,203]^

From a biological point of view, the ability to exert high control over the mechanical properties of hydrogels has been shown promising as it affected cell behavior differentiation.^[69,204]^ For example, they were already successfully used as culture system for myoblasts^[205]^ and organoid development.^[206]^ The Anseth group demonstrated that a PEG-based hydrogel containing telechelic hydrazone crosslinks, which displayed stress relaxation properties close to the native tissue, stimulated chondrocytes to produce matrix^[207]^ and directed MSC toward the chondrogenic lineage.^[160,208]^ The ability to tune the mechanical properties of hydrogels make these dynamic covalent systems interesting for further use in OC applications.

## 5 Conclusions

When regeneration of the OC tissue interface is aimed for, designing scaffolds containing gradients of physical and/or biological properties is required to direct cell fate heterogeneously. This review highlighted the state-of-the-art developments in polymeric scaffolds prepared by AM technologies in view of osteochondral regeneration. We identified that most of the scaffolds so far developed contained discrete gradients, but recent studies also started to report the design of scaffolds with continuous gradients, which hold the potential to better mimic the physio-chemical, mechanical, and biological gradients that characterize native tissues. The fabrication of hybrid —a combination of hydrogel and solid materials— AM scaffolds is being increasingly proposed as a route toward better replicating the soft-hard interfaces present in the OC unit. From a biomimetic point of view, these trends seem promising and may enable successful regeneration of the OC interface. Recommendations are provided to tackle remaining challenges like the use of materials that contribute to the dynamic nature of the tissue. Therefore, we finally discussed advances in the development of dynamic materials to improve AM scaffolds applied for OC regeneration. Although capturing the unparalleled in-vivo complexity remains challenging, scaffold design for OC applications is advancing toward higher levels of biomimicry.
